# Multi-Scale Measures of Rugosity, Slope and Aspect from Benthic Stereo Image Reconstructions

**DOI:** 10.1371/journal.pone.0050440

**Published:** 2012-12-12

**Authors:** Ariell Friedman, Oscar Pizarro, Stefan B. Williams, Matthew Johnson-Roberson

**Affiliations:** Australian Centre for Field Robotics, University of Sydney, Australia; The Australian National University, Australia

## Abstract

This paper demonstrates how multi-scale measures of rugosity, slope and aspect can be derived from fine-scale bathymetric reconstructions created from geo-referenced stereo imagery. We generate three-dimensional reconstructions over large spatial scales using data collected by Autonomous Underwater Vehicles (AUVs), Remotely Operated Vehicles (ROVs), manned submersibles and diver-held imaging systems. We propose a new method for calculating rugosity in a Delaunay triangulated surface mesh by projecting areas onto the plane of best fit using Principal Component Analysis (PCA). Slope and aspect can be calculated with very little extra effort, and fitting a plane serves to decouple rugosity from slope. We compare the results of the virtual terrain complexity calculations with experimental results using conventional *in-situ* measurement methods. We show that performing calculations over a digital terrain reconstruction is more flexible, robust and easily repeatable. In addition, the method is non-contact and provides much less environmental impact compared to traditional survey techniques. For diver-based surveys, the time underwater needed to collect rugosity data is significantly reduced and, being a technique based on images, it is possible to use robotic platforms that can operate beyond diver depths. Measurements can be calculated exhaustively at multiple scales for surveys with tens of thousands of images covering thousands of square metres. The technique is demonstrated on data gathered by a diver-rig and an AUV, on small single-transect surveys and on a larger, dense survey that covers over 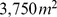. Stereo images provide 3D structure as well as visual appearance, which could potentially feed into automated classification techniques. Our multi-scale rugosity, slope and aspect measures have already been adopted in a number of marine science studies. This paper presents a detailed description of the method and thoroughly validates it against traditional *in-situ* measurements.

## Introduction

Terrain complexity is strongly correlated to biodiversity in marine environments [1]–[4]. Even when terrain is represented as digital bathymetry, it is necessary to abstract these digital terrain models into simpler representations in order to perform analytical work. Ecologists typically use indices, such as rugosity, slope and aspect to describe habitat structure [5]. Rugosity is a measurement that provides a notion of terrain complexity. It is a ratio between the actual length (or area) along the undulating terrain and the straight-line distance (or planar projected area). Values of 1 typically indicate flat terrain and the higher the complexity of the terrain, the higher the rugosity value. Fine-scale rugosity is traditionally measured *in-situ* by divers along a single, linear profile using chain-tape methods [1], [6], [7] or profile gauges [1]. In these methods, rugosity is calculated to be the ratio between the length of the contoured surface profile and the linear distance between the end points. These traditional methods are labour intensive, depth limited and put humans at risk. As a result, surveys tend to be spatially and temporally sparse and not easily repeatable. These measurements are performed using scuba, usually at depths of less than 30 m, which means that the majority of marine habitats cannot be described by this measure. Furthermore, the outputs of transects using the traditional approach are calculated at a single, predefined resolution and scale imposed by the link-size (or gauge spacing) and the transect length. This is an important limitation since some spatial patterns and processes operate at scales not well resolved by the particular choice of chain or gauge [7]. In addition, using a length measure to capture 3D structure is not well suited to characterise the holistic features of natural landscapes [4], and measurements are prone to dramatic variation with minor changes in chain placement. When handling a physical chain *in-situ*, it may be difficult to lay out in a perfectly straight line from start to end, and this may lead to an over estimate of the rugosity due to side-to-side variation in the chain's path. Draping a chain also has an environmental impact that may lead to modifying or damaging the survey site.

Performing virtual calculations over georeferenced, high-resolution 3D bathymetry deals with these issues. It is also possible to perform calculations that better account for the 3D nature of the terrain in ways that would be impossible to measure in the field. The methods have little to no environmental impact, can be easily repeated for monitoring purposes and can be computed at multiple scales over large spatial extents.

There has been previous work that derives terrain complexity measures from bathymetric maps collected from ship borne surveys [8], [9]. However, these methods cannot resolve fine scale structure due to the resolution of the survey data. Other studies have used airborne LIDAR to measure topography [10], but unfortunately these measurements are depth limited due to the poor penetration of the laser in water. In addition, neither of these techniques capture a representation that is easy to interpret visually.

Underwater vehicles, capable of high precision navigation, and equipped with downward-looking stereo cameras can recover bathymetry at fine resolutions over relatively large, contiguous extents of seafloor [11]. Measures derived from these surveys make it possible to obtain dense coverage over larger spatial extents and beyond the depths safely attainable by human divers [12]. Given that the surveys and calculations can be performed without humans, a potential source of measurement bias is eliminated. Furthermore, Autonomous Underwater Vehicles (AUVs) with proper navigation systems provide the ability for easy repeat transects, making it possible to revisit an area of interest for monitoring purposes [11].

Rugosity for a 3D surface is defined as the ratio between the area of the contoured or draped surface and the area of its orthogonal projection onto a plane. A method for calculating rugosity on raster-formatted digital elevation grids has been proposed by Jenness in [5], however forcing an irregular mesh into a raster grid causes reconstructions to be less accurate. Furthermore, Jenness's proposed rugosity calculation is subject to edge-effect problems and by using the horizontal planimetric area, rugosity is affected by slope.

The method proposed in this paper uses the geo-referenced stereo imagery obtained using AUVs or a diver-held stereo-camera rig to generate fine-scale bathymetric reconstructions with centimetre resolution in the form of irregular 3D triangular meshes [12]. Unlike a real chain, conducting measurements on a virtual surface allows for the measurement of complex features such as overhangs and underhangs. It may, however, be useful to note that the downward-looking stereo cameras that were used for this paper, collected imagery from a bird's eye view, with an altitude on the order of 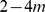. As a result, the terrain reconstructions that we are working with did not generally capture the structure of these occluded features, but with a multi-view camera setup, these measurements would be possible. The use of image-derived bathymetry also provides the potential to combine interpretations based on 3D structure and visual appearance, which has proven useful for deriving descriptors for automated classification of benthic imagery [13]–[16]. We propose a new method for calculating rugosity, derived from the sum of the area of the triangles that make up the surface and dividing that by the sum of their projections onto the plane of best fit. Fitting a plane to the data ensures that rugosity and slope are decoupled at the scale of the chosen window size. As a consequence of fitting a plane, obtaining slope and aspect is trivial.

There are already a number of ecological and biological studies that have made use of our fine-scale measures of terrain complexity [13], [17], [18]. This paper builds upon our previous publication [15] and provides a detailed explanation of the calculations, presents multi-scale results on real data and validates the results using an experiment designed to compare our method to the traditional *in-situ* chain-tape survey technique. The code used in this paper can be downloaded from:

http://marine.acfr.usyd.edu.au/permlinks/afri7947/code-trisurfterrainfeats.php.

The remainder of this paper is organised as follows. sec:data outlines the stereo imaging platforms and the data processing pipeline. sec:calcs presents a detailed explanation of how measurements of rugosity, slope and aspect can be calculated from the stereo-derived 3D meshes. sec:validation provides validation results comparing traditional *in-situ* measured chain-tape measurements to equivalent virtual chain-tape and area-based calculations conducted on the 3D reconstructions. We also present results for surveys performed by a diver-rig and an AUV, and then finally sec:conclusion shows conclusions and presents directions for future work.

## Materials

**Figure 1:**
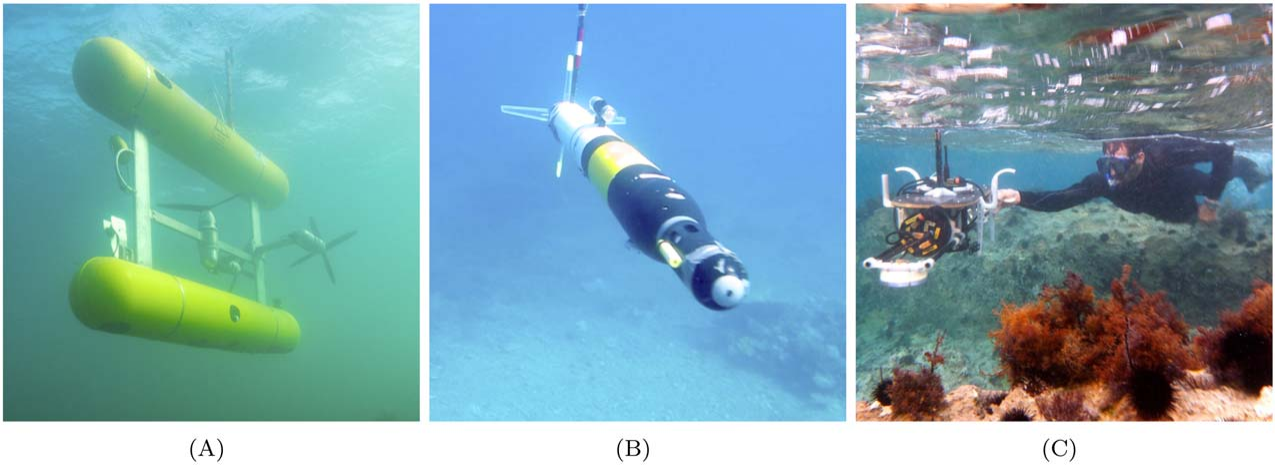
ACFR stereo imaging platforms in action. (A) shows *Sirius* AUV, (B) shows *Iver2* AUV and (C) shows the diver-rig.

The University of Sydney's Australian Centre for Field Robotics (ACFR) develops and operates underwater stereo imaging systems that have been used on a selection of AUVs, Remotely Operated Vehicles (ROVs) [19], manned submersibles and diver-held systems [20]. Photos of example platforms are shown in Figure 1. While AUVs (Figure 1(A) & (B)) are capable of comparatively large spatial coverage, the diver-rig (Figure 1(C)) is useful for performing rapid surveys in shallow water without the need for any additional infrastructure or ship time.

The platforms are all designed for high-resolution, georeferenced survey work and each includes a downward-looking camera pair with a baseline of approximately 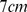, pixel resolution of 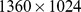 and a field of view of 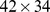 degrees. The platforms carry their own light and power sources and typically aim to maintain an altitude of 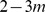, capturing overlapping stereo image pairs at a frequency of 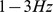, depending on platform speed and altitude. This results in 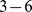 views of each scene point. All of the platforms have a suite of navigation sensors including GPS (for the surface), a pressure/depth sensor, a compass and inclinometers. The AUVs and ROVs are usually also fitted with Doppler Velocity Logs (DVL) and Ultra Short Baseline (USBL) transponders as well a selection of oceanographic and acoustic sensors.

Using the visual-aided navigation pipeline from [21] and the meshing system described in [20], the stereo imagery is combined with pose estimates to deliver fine-scale 3D, texture mapped terrain reconstructions. The processing pipeline for generating the stereo meshes is broken down into the following steps:

1. **Data Acquisition and Preprocessing:** The stereo imagery is acquired by a stereo-imaging platform and preprocessed to partially compensate for vignetting, lighting and wavelength-dependent colour absorption [20].
2. **Visual SLAM:** The platform poses are estimated through a technique called visual Simultaneous Localisation and Mapping (SLAM) [21]. Images are searched for visual loop closures and all the data from various navigational sensors are fused together to make a consistent estimate of the platforms's pose and location at every instant a stereo photo pair is captured. A visual loop closure can be thought of as a recognised landmark identified from the images. When a landmark is observed for a second time, it is possible to correct the estimated platform position to improve its navigation solution.
3. **Stereo Depth Estimation:** 2D features are matched between stereo image pairs and the 3D position is determined by triangulation. The 3D point clouds are converted into Delaunay triangulated meshes.
4. **Mesh Aggregation:** The individual stereo meshes are put into a common reference frame using SLAM-based poses and fused into a single mesh using volumetric range image processing (VRIP) [22]. Discontinuities between integrated meshes are minimised and simplified versions of the mesh are produced to allow for fast visualization at broad scales. The average resolution of the simplified 3D mesh is 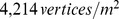, with an average triangle edge length of 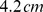.
5. **Texturing:** The polygons of the complete mesh are assigned textures based on the projection of overlapping imagery, and the result is a large-scale photo-realistic 3D reconstruction of the benthos [12].

## Methods

The digital terrain reconstruction is defined by a Delaunay Triangular Irregular Network (TIN) which is made up by a set of triangular faces that connect vertices to make a 3D surface [23]. The vertices of the surface are contained in the set 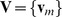, such that 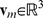 and 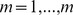, where 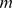 is the total number of vertices in the surface. 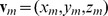 represents the vertex 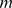 described by its 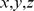 coordinates. The triangles of the surface are contained in the set 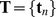, where 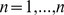, such that 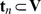, and 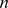 is the total number of triangles contained in the surface. 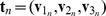 represents a triangle defined by three vertices in 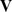.

### Virtual chain-tape rugosity

**Figure 2:**
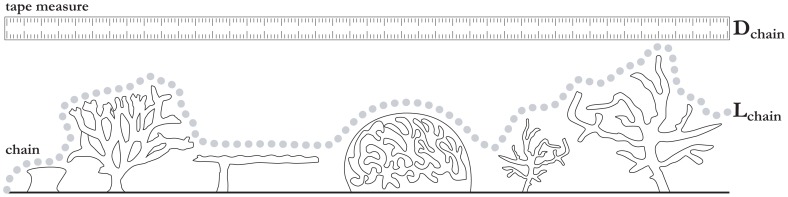
Chain-tape rugosity illustration. Image adapted from [26].

For traditional *in-situ* rugosity assessments, a chain of known length, 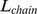, is draped over the undulating substrate in a straight line and the linear distance, 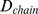, between the end points of the chain is measured using a tape measure, as illustrated by Figure 2.

Rugosity, 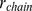, for that transect is then computed to be the ratio between 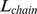 and 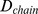, i.e.:
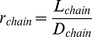
(1)


The rugosity value can vary depending on the resolution and type of chain that is used, however, it will always be a function of terrain complexity. For a flat area, we would expect 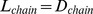 with 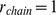. For more complex terrain, 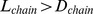 and therefore 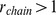.

**Figure 3:**
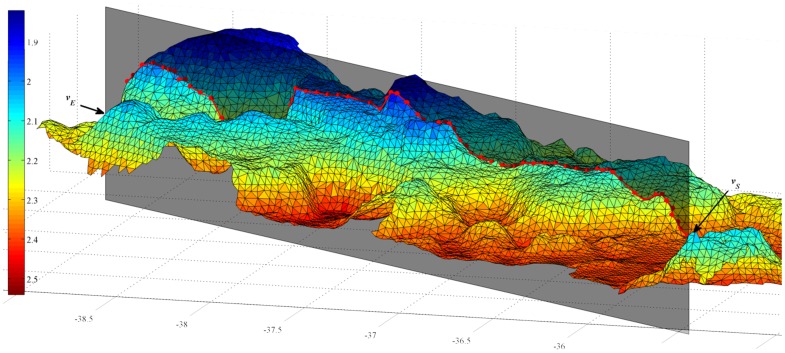
Example of a virtual chain ‘draped’ over a 3D terrain reconstruction. The coloured surface represents the terrain to be examined. The horizontal axis shows Easting (metres) and the colour bar shows depth (metres). The shaded grey plane represents the plane on which the linear rugosity will be measured while the red line and dots represent the ‘chain’, which is made up of those points that fall within a distance of 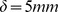 from the plane. The points 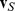 and 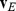 show the start and end verticies of the virtual chain.

Using the reconstructed fine-scale terrain model it is possible to perform virtual chain-tape measures over the TIN. This can be done by specifying three points to define a vertical plane and linking all the vertices in the mesh that lie on (or very close to) the plane to make a virtual chain. Let the plane be defined by a starting vertex, 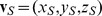, an ending vertex, 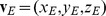 and a third vertex directly above one of the others to define a vertical plane 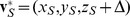, where 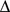 is some arbitrary non-zero value and 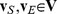. We then define the subset of vertices that make up the virtual chain as, 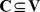. The subset 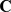 is determined by examining the point to plane distance 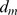 for every vertex in 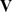 and selecting the ones that fall within a threshold, 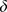, to the plane. The value of 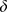 needs to be selected based on the resolution of the mesh and the point-plane distance is given by the equation,
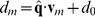
(2)where 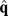 is the unit vector normal to the plane and 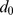 is the distance of the plane from the origin. The normal vector can be found by taking the normalised cross product of two vectors that lie on the plane:
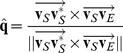
and 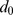 is a constant that can be calculated from 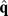 and a point on the plane, e.g.:
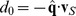



We can then compute the Euclidean distance matrix for all the vertices in 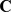 and starting at 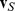, we trace out a virtual chain by linking all the adjacent vertices in one direction until we reach 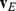. An example of this is shown in Figure 3.

The virtual chain-tape rugosity in equ:chainrgsty can then be computed by dividing the sum of all distances between the adjacent vertices in 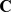, to give 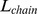, and dividing it by 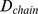 which is simply the straight-line Euclidean distance between 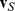 and 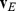.

### Virtual area-based rugosity

Given that we have a 3D reconstruction of the terrain, we can compute a ratio of areas, as opposed to a ratio of lengths. The rugosity index for a particular location in the terrain mesh can be calculated by dividing the surface area of the undulating terrain by the area of the orthogonal projection of the surface onto a plane. Instead of selecting the length of the chain, we select the size and shape of the bounding box or window with which to do the calculation. The area-based rugosity index, 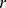, is therefore:
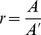
(3)where 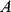 is the surface area of the undulating terrain within the window, and 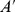 is the area of the orthogonal projection of that surface onto a plane.

The window can be described by the subset of triangles and vertices that it encloses. The subset of vertices are contained in 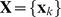, such that 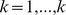 and 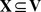, where 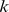 is total number of vertices that are contained within the window. A vertex is only included in 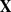 if it forms part of a triangle that falls entirely within the window. The subset of triangles within the window are contained in 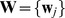, where 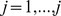 and 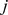 is the total number of triangles that are contained in the window. 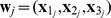 represents a triangle comprised of three vertices in 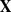, such that 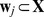.

The area of the contoured surface bounded by the window 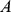, is equal to the summation of the areas of all the individual triangles that are contained within the window
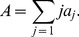
(4)


The area of an individual triangle, 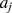, in the contoured surface can be calculated to be half the magnitude of the cross product of the vectors representing two adjacent sides of the triangle. The intuition for this calculation is as follows: let a triangle in the surface, 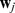, be defined by the vertices 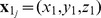, 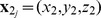, 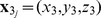, and the adjacent vectors 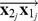 and 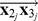 to be:
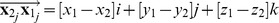


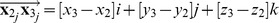
The area of a parallelogram with sides 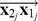 and 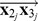 is equal to the magnitude of the cross product of vectors representing two adjacent sides. The area of an individual triangle 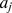 is then half of this, and can be expressed as
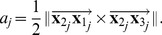
(5)


Next we need to consider the projected area 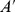, which is the area of the orthogonal projection of the surface contained within the window, onto a plane. The correct choice of plane is an important consideration. Simply projecting the points onto the horizontal 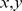 plane by setting the 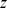 components to zero, for example, confounds the rugosity measurement by coupling it with slope. This would mean that flat, steep terrain would exhibit an overstated rugosity index. Ideally, we would like to have rugosity decoupled from slope at the scale of the chosen window size. Therefore, we require the area of the orthogonal projection of the surface onto the plane that best fits its vertices (contained in 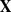). The plane that best represents the data can be obtained using Principal Component Analysis (PCA).

PCA is used to determine the orthogonal projection of the data onto the *principal subspace* (a lower dimensional linear space) such that the variance of the projected data is maximized [24]. It involves evaluating the mean and the covariance matrix of the data 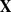 and then finding the eigenvectors and corresponding eigenvalues of the covariance matrix. By ordering the eigenvectors in the order of descending eigenvalues, an ordered orthogonal basis 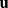 is created containing the eigenvectors
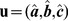
(6)where 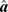 is the principal component and has the direction of largest variance of the data, 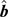 is the secondary component, and 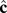 is the third component and has the direction of the least variance of the data, and is orthogonal to the principal and secondary components. Consequently, 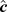 is a direction vector normal to the principal plane of the data, however, it is ambiguous as to whether it is inward or outward facing. Given that the data is obtained from overhead imagery, it is assumed that the outward facing normal will always have an upward facing component. This is enforced by checking the sign of the dot product between 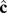 and the upward facing unit vector, 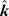


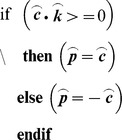
where, 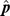 is the outward-facing normal to the principal plane of the data.

The projected area 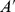 can now be expressed as a summation of the areas of the individually projected triangles bound by the window
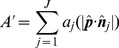
(7)where
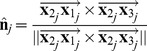
is the unit vector normal to the face of triangle 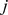 and 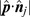 gives a ratio for the projected area of the triangle on the plane to its actual contoured area in 3D space. From this, it is possible to compute the rugosity index shown in Equation 3.

### Other virtual terrain measurements

Given that we now have the vector, 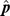, normal to the plane of best fit, it is relatively straightforward to obtain measurements for the slope and aspect of the same windowed region of the terrain.

#### Slope

Slope, denoted by 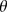, refers to the angle between the plane of best fit and the horizontal plane. This angle is equivalent to the angle between the normal vectors of the two planes and can be obtained from their dot product, which is 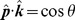 (noting that 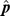 and 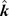 are both unit vectors). Thus, slope can be calculated as
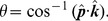
(8)The slope is a positive angle in the range 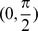.

#### Aspect

Aspect, denoted by 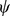, refers to the direction that the surface slope faces. It is defined as the angle between the positive 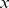 axis and the projection of the normal onto the 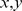 plane. It can be calculated as
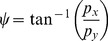
(9)where 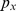 and 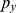 are the components of 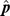 in the 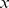 and 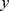 directions, respectively, and 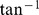, in this case, is the 4-quadrant inverse tangent that outputs an angle in the range 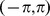. For analytical purposes, it may be useful to split aspect into vector components to eliminate the discontinuity associated with angular wrap-around:
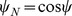


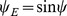
where 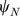 denotes ‘Northness’ and 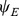 denotes ‘Eastness’.

## Results

In this section, we compare the virtual measurements obtained from the reconstructed terrain models to traditional *in-situ* measurement techniques, and we also present results for real data collected by a diver-rig and an AUV. Except for the *in-situ* experiments that used a physical chain, the methods proposed in this study are completely non-contact and do not require physical samples to be collected. The *in-situ* experiments did not involve endangered species or protected areas, and accordingly, no specific permits were required for the described field studies.

### Field validation experiment

**Figure 4:**
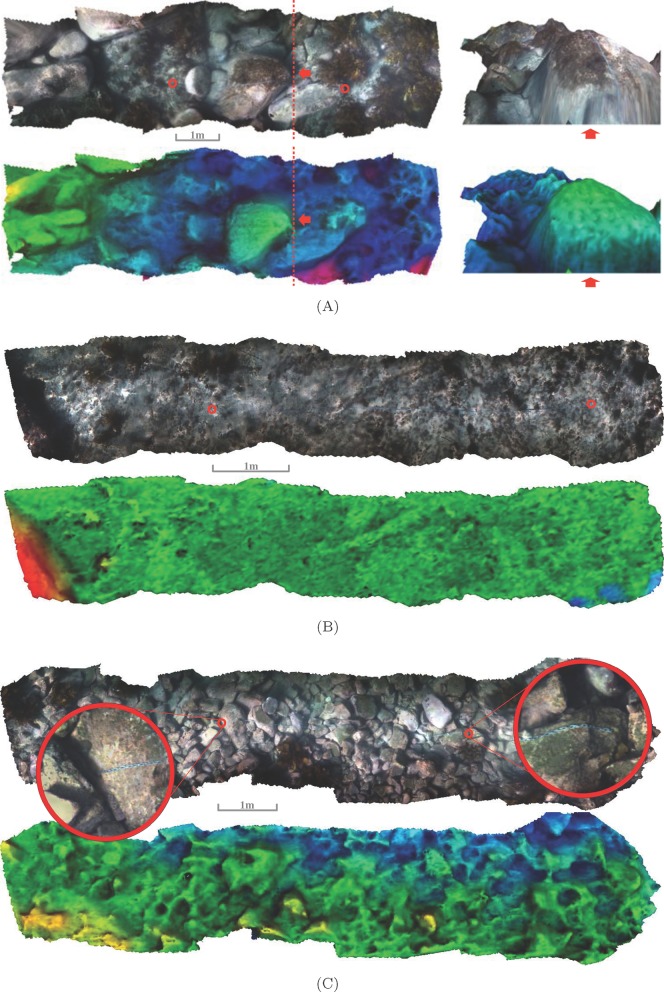
Example survey transects showing different bottom types. The figures show the photo-realistic 3D mosaic and also the depth mapped bathymetry for each transect. The small red circles show the start and end points of the chain (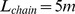) that was laid out over the terrain. (A) shows a highly rugged patch (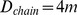, 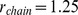). It also shows the same patch from an oblique perspective. (B) shows a relatively flat patch (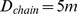, 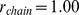) and (C) shows a patch with medium relief (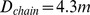, 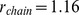). There is also a zoomed in view of the start and end of the chain shown in (C).

We carried out an experiment that involved laying down and measuring a physical chain (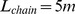) over a selection of different transects with varying bottom types. We then surveyed each transect with the diver-held stereo imaging platform, shown in Figure 1(C). After processing the data and generating the georeferenced photo-realistic 3D meshes, we were able to pick out the locations of the start and end points of the chain for each transect and then calculate the virtual chain-tape measure explained in sec:virtchain. Figure 4 shows example transects, and Figure 4(C) shows a zoomed in view of the start and end points of the chain. The location of these points was used as the start and end points for draping the virtual chain.

**Figure 5:**
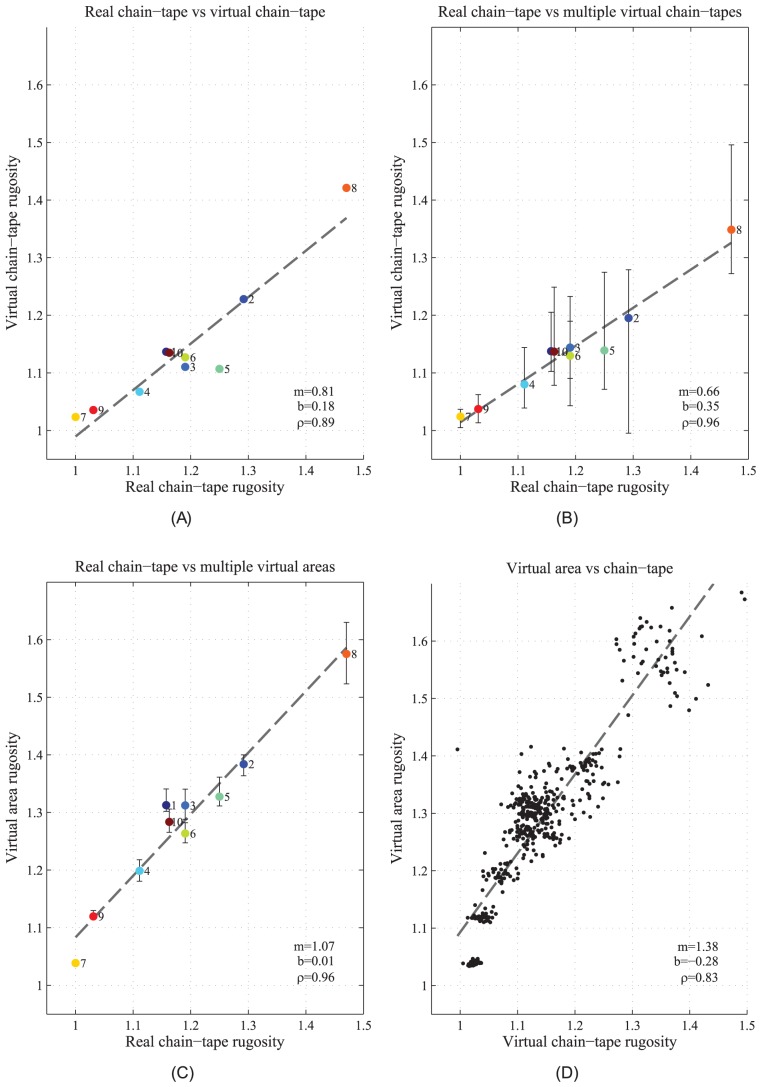
Comparison of virtual and *in-situ* measured rugosity measurements. (A) shows virtual chain rugosity values vs physical chain rugosity measurements for 10 different transects with varied bottom types. (B) shows the mean, minimum and maximum virtual chain-tape rugosity values for 49 virtual chains translated by less than 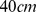 from the measured location for each of the 10 transects vs the physical, real chain-tape rugosity measurements. (C) shows the mean, minimum and maximum virtual area-based rugosity with 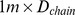 sized windows centred and oriented over the 49 virtual chains for each of the 10 transects vs the physical, real chain-tape rugosity measurements. (D) compares each virtual chain-tape rugosity to the corresponding virtual area-based rugosity for all 490 virtual measurements (49 for each of the 10 transects.) The figures also show the least-squares linear regression fit of the means, 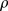: correlation, 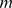: slope and 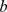: intercept per transect.

Figure 5(A) shows the virtual chain rugosity measures vs the physical *in-situ* chain rugosity measurements for 10 different transects with varied bottom types. It shows a correlation of about 0.89 between the two measurements. The slope of the line of best fit to the data is 0.81 suggesting the real chain-tape rugosity values are generally higher. Explanations for this may be attributable to the fact that it is quite difficult to lay the chain out in a perfectly straight line when out in the field. Side-to-side variations in the real chain's placement may cause the rugosity to be overestimated. In addition slop in the real chain's links may lead to the chain bunching up in places, which would also cause the *in-situ* chain rugosity measurement to be overestimated.

The results in Figure 5(A) show that it is possible to obtain similar measurements from the reconstructions to what divers would recover out in the field, but without any chains and tapes. This method also allows greater flexibility with regards to the size and positioning of the ‘chain’ and it is possible to acquire this data using machines without putting humans at risk. In addition, the reconstructions constitute a visual record of the surveyed transect.

**Figure 6:**
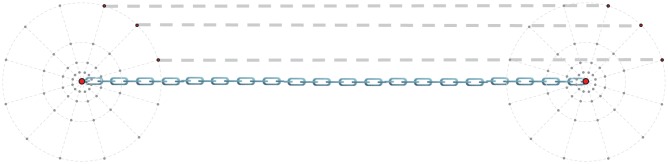
Illustration showing systematic translation of virtual chain placement. The start and end points of the chain were moved from the original measured locations by 5 cm, 10 cm, 20 cm and 40 cm, at 12 different points spanning a full circle with 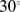 increments. This results in a total of 49 virtual chains per transect, all with similar length and orientation. The figure shows the original measured chain positions (big red points in centre of circles), and three examples of the 48 additional translated virtual chains connecting the corresponding start and end points.

In an attempt to determine how much the results vary with minor changes to chain placement, we translated the virtual chain position by varying its start and end locations by a small amount, keeping the chain orientation and measured length, 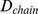, constant. The start and end points of the virtual chains were translated about the original measured locations by 5 cm, 10 cm, 20 cm and 40 cm, at 12 different points spanning a full circle with 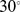 increments (i.e.: it is moved around in a manner similar to the coupling rod connecting the wheels of a train). This results in 48 additional chains per transect, all ‘laid out’ in parallel with the same orientation, but with minor translations in positioning. Figure 6 illustrates how the virtual chain was translated about the terrain reconstruction.

Figure 5(B) shows the mean, minimum and maximum rugosity values for the 49 virtual chains translated about the same transect. The mean rugosity values of the 49 virtual chains translated about the measured start and end points exhibit an even stronger correlation with the physical chain measurements, of 0.96 (for the means). However, there is a large spread between the minimum and maximum virtual chain-tape rugosity values over each transect. The virtual chain-tape rugosity index varied as much as 0.28 on a single transect which equates to a difference of 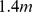 in the straight line measurements, 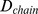. This large variation due to minor changes in virtual chain placement (of less than 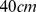), suggests that a 1D length measure may not be well suited to capture 3D terrain structure and it motivates the need for a measure that is more robust to minor variations in positioning. A 2D area-based measurement of rugosity is less sensitive to this because with small changes in positioning, most of the area within the window is still over the same terrain, compared to the chain that may be draped over completely different terrain features. Consequently, the area based rugosity measurement is a more representative measure of the terrain complexity. Figure 5(C) shows the results of the real chain-tape rugosity vs virtual area based rugosity for 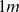-wide windows centred over the 49 virtual chains, with the lengths and orientations of the windows the same as that of the virtual chains. Even though these measurements are quite different, it is apparent that a strong correlation still exists between the rugosity values for the area-based measurement and the real chain-tape measures (0.96 for the means). However, the area based measurement is taking the structural complexity of a 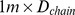 window into account, and it is apparent that it is far more robust to changes in placement and therefore more repeatable, with a much lower spread between the minimum and maximum values resulting from translating the window over the transect, when compared to translating the virtual chain. Figure 5(D) shows a plot comparing virtual chain rugosity to virtual area rugosity. It shows an increase in variability with increasing rugosity.

### Small-scale, single transect diver-rig survey results

**Figure 7:**
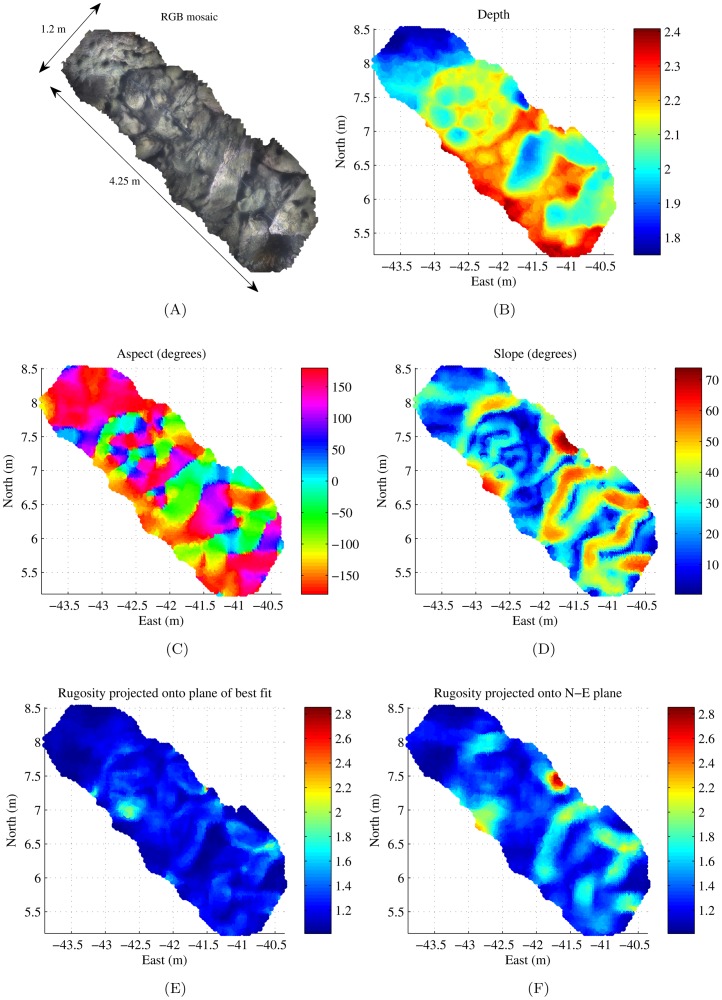
Fine-scale surface complexity measurements for a small, single transect diver-rig survey. Results were computed with a window size of 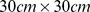 positioned over every vertex in the mesh. (A) shows the photo-realistic 3D mosaic, (B) shows the depth/bathymetry map, (C) shows aspect, (D) shows slope, (F) shows area-based rugosity projected onto the N-E plane and (E) shows rugosity projected onto the plane of best fit.

The diver-rig can be used to obtain dense reconstructions of a patch of interest, or reconstructions along a single transect, as shown in Figure 4. It is a useful tool for rapid diver-based assessments and does not need the supporting infrastructure required by an AUV or ROV. Figure 7 shows results for a diver-rig survey conducted in Fairlight, New South Wales, Australia. It consists of a single transect spanning approximately 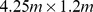. Figure 7(A) shows an overhead view of the 3D photo-realistic mosaic and Figure 7(B) shows the bathymetry/depth map. The results in Figure 7(C)–(F) show results for aspect, slope and rugosity calculated at a resolution of 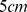 with a relatively small window size of 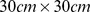.

#### The effects of projecting to the plane of best fit

From Figures 7(D) and (F), it is apparent that the rugosity projected onto the N-E horizontal plane appears to be higher at regions of higher slope. Comparison of Figures 7(E) and (F) highlights the effect of projecting the area onto the plane of best fit.

**Figure 8:**
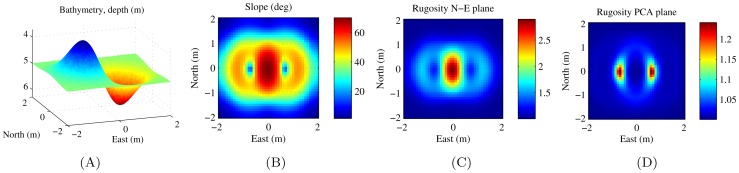
Results for simulated terrain model for exponential function. 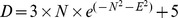
, where 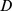, 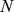 and 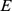 are Depth, Northing and Easting in metres. The results are computed with a mesh resolution of 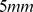 and a window size of 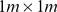. (A) shows an oblique view of the 3D bathymetry, (B) shows the slope angle, (C) shows the rugosity projected onto the N-E horizontal plane and (D) shows the rugosity projected onto the plane of best fit.

In order to provide an understanding of the results, we ran the calculations on a simple simulated terrain example made up of a peak and a trough with a point of inflection between them that has a high slope. Figure 8 shows results for a simulated surface. From Figures 8(B) and (C) it is apparent that the rugosity projected onto the N-E horizontal plane is highest at the point of maximum slope. Figure 8(D) shows the rugosity projected onto the plane of best fit (PCA plane), and shows the highest values at the stationary points, which are points of zero slope.

This decoupling with slope is supported by examining the correlation matrices for the different calculations. tab:corrmatfairlight shows the correlation matrix for the diver-rig survey and tab:simsurfcorrmat shows the correlation results for the simulated terrain. In both cases we can see that slope angle and the values for rugosity projected onto the N-E horizontal plane are very strongly correlated, and although there is still a mild correlation between slope and PCA plane rugosity, there is a stronger correlation between PCA plane rugosity and N-E horizontal plane rugosity. It is apparent that fitting a plane serves to decouple rugosity from slope.

### Broad-scale, dense AUV survey results

**Figure 9:**
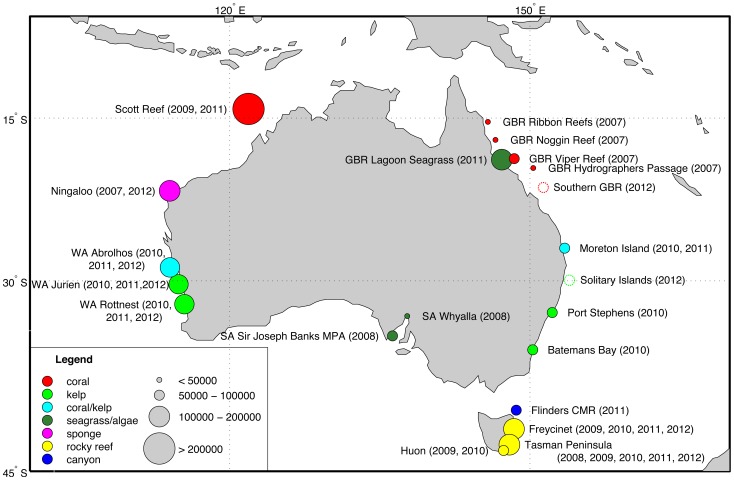
AUV survey locations around Australia [11]. The circles are coloured by dominant habitat type and scaled based on the number of images currently available in the IMOS AUV Facility image archive.

**Figure 10:**
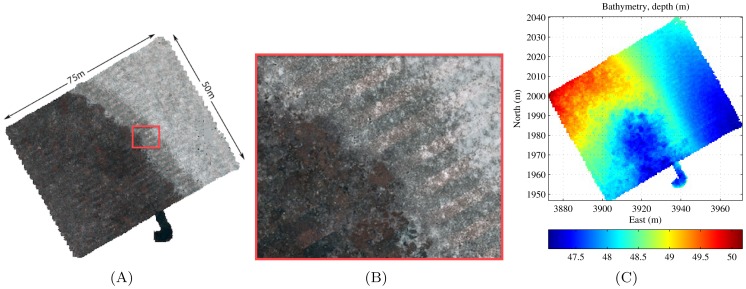
Dense AUV grid at Scott Reef off western Australia covering 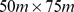 with 9,831 stereo image pairs. (A) Textured 3D mesh overview of survey site reconstructed using the method outlined in sec:data. (B) Close up of transition zone showing dense coral cover, barren sand and an intermediate, partially populated substrate class. (C) Colour map of mesh depth/bathymetry.

The AUV *Sirius* is part of the Integrated Marine Observing System (IMOS) and is used to collect repeatable, time-series data at various sites around Australia [11]. Figure 9 outlines the current repeat monitoring sites and provides a sense of the scale of the AUV observing program. Figure 10 shows the results for an AUV survey performed at Scott Reef that densely covered an area of 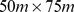 with 9,831 stereo image pairs. This survey featured a partially populated substrate boundary between dense coral and barren sand, as illustrated by Figure 10(B).

**Figure 11:**
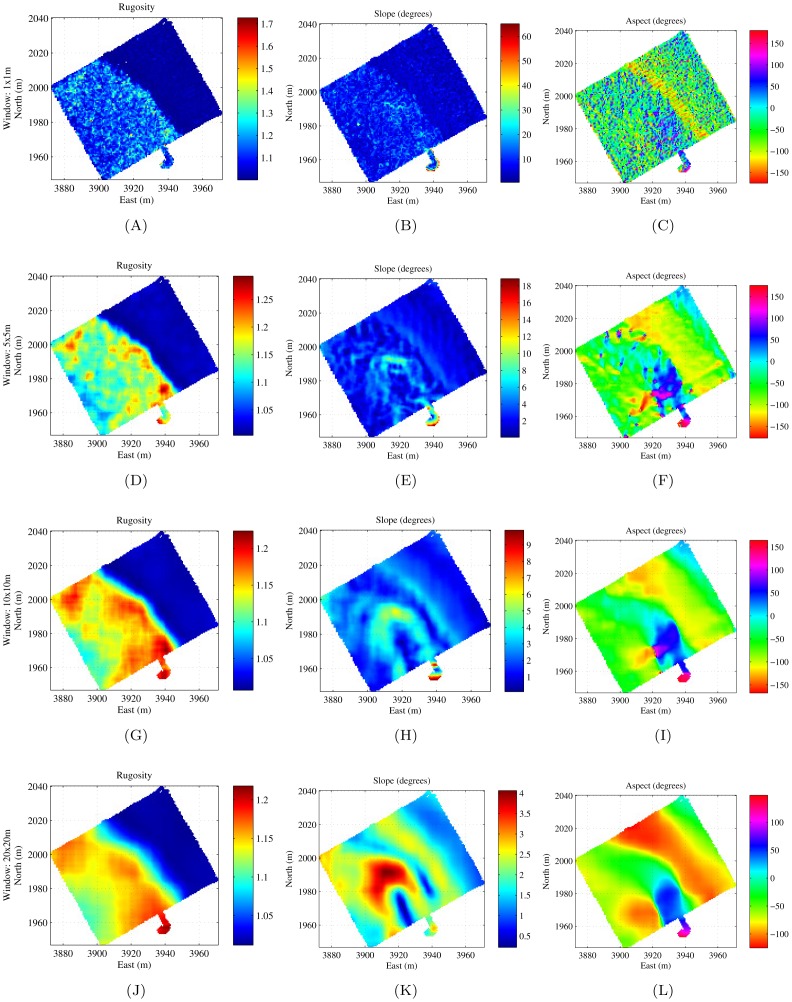
Dense AUV grid completed in Scott Reef showing the effect of different window sizes on the results. (A), (B) and (C) show rugosity, slope and aspect with a window of 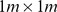. (D), (E) and (F) show rugosity, slope and aspect with a window of 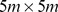. (G), (H) and (I) show rugosity, slope and aspect with a window of 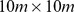. (J), (K) and (L) show rugosity, slope and aspect with a window of 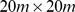.

Figure 11 shows the effect of different window sizes on the calculation of rugosity, slope and aspect. A larger window provides more spatial smoothing, however too much smoothing causes information loss. It can be seen from Figure 11 that rugosity appears to be a good indicator for the different substrate types and it outlines the boundary between the different substrates shown in Figures 10(A) and (B) quite closely. Consequently, these measures have been found to be useful descriptors for automatically discriminating different habitat types [13]–[16].

#### A note on aspect angle

The aspect angle must be considered with reference to the slope, i.e. at regions where the slope is close to zero, the aspect is relatively erratic since the normal vector points almost directly up and the direction of the component of the normal projected onto the N-E plane, changes dramatically with a small change in any of the variables in the calculation. It should also be noticed that aspect is subject to angular wraparound where a value of 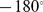 should be interpreted to be the same as a value of 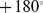. This needs to be taken into consideration when interpreting the results. Consequently in Figures 7 and 11, the aspect plots were displayed using a circular colour map that shows a continuos blend about 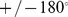. Measurements of aspect are likely to be more useful for classification purposes when framed in context with water currents and environmental conditions to calculate a notion of exposure. It is also possible to weight aspect with slope angle to provide a notion of magnitude.

#### Effects of window size

The window size needs to be chosen with reference to the spatial scales of the environmental features to be considered. It can be likened to the chain/transect length in the conventional chain-tape method, of which the importance of scale has been outlined in [2], [3], [25]. The window size has an impact on the discriminatory power of the measure as a descriptor. Smaller window sizes do not capture as much variation in the ruggedness of the surface and larger window sizes provide spatial smoothing of the results. This is demonstrated by the results in Figure 11. The window size needs to be selected in accordance with the scale of processes to be observed.

#### Effects of mesh resolution

The mesh resolution is analogous to the link-size for the chain-tape method. The importance of link size is explored in [7]. Over the experiments that we performed, coarse mesh resolutions impacted the accuracy of the results, particularly with small window sizes. Resolutions that are too fine may be susceptible to noise in real-world terrain reconstructions that arises from uncertainty in the 2D feature locations and in the estimate of the stereo camera calibration parameters. We found that the 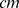-scale mesh resolutions that we typically work with, coupled with window sizes on the order of metres provide repeatable, robust results. It may also be important to note that just as it would be difficult to compare rugosity values computed with different chain link sizes, it may be difficult to compare virtual terrain complexity measurements computed with different mesh resolutions. The resolution should be chosen such that it is robust to noise, while still maintaining an adequate representation of the variability in the terrain.

## Conclusion and Future Work

This paper has demonstrated how multi-scale measures of rugosity, slope and aspect can be derived from fine-scale bathymetric reconstructions created using georeferenced stereo imagery collected by AUVs, ROVs, manned submersibles or diver-held stereo camera systems. We presented a new method for calculating rugosity by considering the area of triangles within a window and their projection onto the plane of best fit, which was found using PCA. Through obtaining the plane of best fit, rugosity is decoupled from slope, and as a consequence of fitting a plane, slope and aspect are calculated with very little extra effort. The results of the virtual terrain complexity calculations were compared to experimental results using conventional *in-situ* measurement methods. It was shown that performing calculations over a digital terrain reconstruction is more robust, flexible and easily repeatable. We showed that using the digital 3D terrain reconstructions, it is possible to perform measurements that are difficult (if not impossible) to obtain manually in the field. In addition, the techniques are completely non-contact, which reduces the environmental impact of the surveying technique, making it more useful for repeat monitoring. Using an autonomous platform, the measurements can be collected without putting a human in the water, and beyond traditional scuba depth limits. The technique was demonstrated on small single transect surveys gathered by a diver-rig and on a larger AUV survey consisting of tens of thousands of images covering thousands of square metres.

Future work may involve combining slope and aspect with current flow fields inferred using an acoustic doppler current profiler (ADCP), which may provide a good indicator of environmental exposure and a proxy for benthic habitat types. Given the method's computational tractability, it may also prove useful as a virtual ‘sensor’ to inform adaptive surveying strategies such as delineating zones of significant change in rugosity (i.e. interface between reef and sand or healthy and damaged reef). As mentioned in the paper, the visual information co-registered with the structural complexity can be used for improved descriptors for automated classification. Although we have done some work feeding these measures into automated interpretation tools, more work is needed to properly showcase the potential of the presented measures for this purpose.

### Tables

Table 1 shows correlation matrix for slope, PCA plane-fit rugosity and horizontal N-E plane rugosity for diver-rig survey. Results were computed with a window size of 30 *cm*×30 *cm*. Table 2 shows correlation matrix for slope, PCA plane-fit rugosity and horizontal N-E plane rugosity for simulated terrain. Results were computed with a resolution of 5 *mm* with a window size of 1 *m*×1 *m*.

**Table 1:** Correlation matrix for slope, PCA plane-fit rugosity and horizontal N-E plane rugosity for diver-rig survey.

	SLOPE	RGSTY-PCA	RGSTY-NE
SLOPE	1	0.21	0.85
RGSTY-PCA	0.21	1	0.56
RGSTY-NE	0.85	0.56	1

Results were computed with a window size of 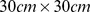.

**Table 2:** Correlation matrix for slope, PCA plane-fit rugosity and horizontal N-E plane rugosity for simulated terrain.

	SLOPE	RGSTY-PCA	RGSTY-NE
SLOPE	1	0.43	0.91
RGSTY-PCA	0.43	1	0.52
RGSTY-NE	0.91	0.52	1

Results were computed with a resolution of 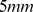 with a window size of 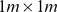.
