# Correction: Multi-Scale Measures of Rugosity, Slope and Aspect from Benthic Stereo Image Reconstructions

**DOI:** 10.1371/annotation/55ee98d1-6731-4bee-81d6-03ce0259c191

**Published:** 2013-12-02

**Authors:** Ariell Friedman, Oscar Pizarro, Stefan B. Williams, Matthew Johnson-Roberson

Due to errors introduced during the production process, some equations do not display correctly.

The upper limit in Equation 4 is not in the correct place. The correct equation can be viewed here: http://plosone.org/corrections/pone.0050440.e004.cn.tif [^]

The "If-Then-Else" statement that follows Equation 6 has incorrect symbols. The correct equation can be viewed here: http://plosone.org/corrections/pone.0050440.004.cn.tif [^]

In the Methods section, in the last paragraph of the subsection "Virtual chain-tape rugosity", the in-text reference for Equation 1 is incorrect. The text reading "equ:chainrgsty" should instead read "Equation 1". 

